# Supplementary material for: Patterns of Intron Gain and Loss in Fungi
Source: PLoS Biol. 2004 Nov 30;2(12):e422. doi: 10.1371/journal.pbio.0020422 (PMC532390; doi:10.1371/journal.pbio.0020422)
Supplement: Table S1 — Also available at http://genes.mit.edu/NielsenEtAl/. (4.3 MB ZIP). [file pbio.0020422.st001.zip › NielsenEtAl/html/1415.html]

AN3371.1.NCU06056.1.MG09244.1.FG09384.1


```
 CLUSTAL W (1.82) Multiple Sequence Alignments - Introns Inserted


Sequence 1: NCU06056.1	591 aa
Sequence 2: MG09244.1	706 aa
Sequence 3: FG09384.1	613 aa
Sequence 4: AN3371.1	589 aa
Alignment Length: 738 aa
Number Identitical Residues: 138 aa
Alignment Score (without introns) 8834


MG09244.1 	MNG0LSRSVSGAWRRWGRRTTTKPHRAERAKQQHQRIRRRISGFRYDERTPHSPAAYRST
NCU06056.1	---~--------------------------------------------------------
FG09384.1 	---~-------------------------------------------------------M
AN3371.1  	---~--------------------------------------------------------
          	                                                            

MG09244.1 	IPHRT0SLVSPTEEAHVPTSTVDDIRIEPRSARTSSDRPPSLHAAADDSSKVA---ATAE
NCU06056.1	-----~-MTPPSDPMHS-----EPLTLDTRLPRAERDQ--SLQEEEEGR-------ATVE
FG09384.1 	DTTDD~GHTGPRAPCPTSVAASALLPIRDGYSPRRTDFGDNRDDDADSDTEFASIMASSM
AN3371.1  	-----~-MTADLLPSSSPFIISTIPPPRPVSVN-----------------------AFAR
          	        .        .                                      *   

MG09244.1 	LASIMTIVSNPVPPGPSSPSLNEAVRPQDFIPPARPSSTPFPQPEPRNAEIKCTSCPAGA
NCU06056.1	LANIMTTASNPVSPGSSSPPLDQAG-LQDFIPPTRPSSTPFPHADPRNATMRRATCPA--
FG09384.1 	LNGIHNTASNPVSPGSSSPSHDQAK-TSDFVPPTRPSSTPFPHPDHRHSKPCCLNGFS--
AN3371.1  	ATSISNTVSCSKSNRSTSAGISSHEGSLTVPVGPDSPGSPQSPVDTEMEDVQSAEPLS--
          	  .* . .* . .  .:*.  ..      .   . ...:* .  : .          :  

MG09244.1 	APHDAHMQGMDAWDDPSFLLQQQPAV-RAGLHFHDLPPEIHECILDHLFGYRVSATSKSS
NCU06056.1	--EDLMDLDEPGHCQPSAGALPGPSQ-SVGVNLNHLPAEIHECILDHLFGFRISPASKS-
FG09384.1 	-----HGADENLAADSAASSDTEPIISHPETNFNDLPIEVHEAILDHLFGYRVSATSRSS
AN3371.1  	-------------------------VCESKVRFEHLPVEIHETILDFLFGERTATITGAS
          	                          .    .:..** *:** ***.*** * :. : ::

MG09244.1 	VGMHSVTKSWSTALRHSRRRELSELALVSSVWRALIQERLYRHI~KIKATVGSVNGALVW
NCU06056.1	----SIVR-WGTALRHSRRRELSELSLVSSVWRILIQERLYRHI~KLKATIESLRGSFSW
FG09384.1 	MAVSSISKSWGTALRHSRRRELTALALVSDIWRVLVQQRLYRHI~KLKATVDCLEDAMVH
AN3371.1  	G--KSSAQSWHKSLRHPRRKALSNLALISPVWRILVQDRIYRHI1KLKGTADELEESARW
          	    *  ::* .:***.**: *: *:*:* :** *:*:*:**** *:*.*   :. :   

MG09244.1 	FAQRPHLRPYVRHVEVWFPVFQP-----------------KPGSPATMVLQATSTLPTVT
NCU06056.1	FCQNPHLRCYVKHIEVWFPVFQP-----------------KYG---PLAVSNTNVLPTVT
FG09384.1 	LAMHEHLQTYVKHIEIWFPVFQP-----------------TYG---PVALSNSLTLPTVT
AN3371.1  	FRAHPHLATYVRHVEIWIPVWGQRAIKNASRQLPRRLNDENAGLTDMAALQATMAWDDPH
          	:  . **  **:*:*:*:**:   : ..:: . .   .... * .   .:. : .     

MG09244.1 	VDGLSASSYVLPVDNCSLDETFYFVGTTFPEVTVLTLEGGERKKAPKVTHFVYGDCTRRA
NCU06056.1	ADGLTNATYLLPGNNCSLEEAFYFVAETFPEVSVLTLEGGERKKAPKVRHWIR-------
FG09384.1 	MEGLTNATYTLPGNNCTLEQVFQFVGQTLPQTRVLTLEGGERRKAPRVLHFPE-------
AN3371.1  	SNPVADYKYYYASHNATLEEMFLHVQTVFRGARMLTLEGGHCKKPPMVRHFKT-------
          	 : ::  .*  . .*.:*:: * .*  .:  . :******. :*.* * *:         

MG09244.1 	QEASSQYSPFWESTVSENVERIMPKVPSVRTLVCKGQWNLIRSEEDFQHIMSALPNLQEW
NCU06056.1	-EERGQ-------------LRAMPKVETIRTLICKGQWNLIRSLQDWEAIMSALPNLQEW
FG09384.1 	-QRIDP-----------AIYKSLPTLHSVQTLVTRGQWNLMRDNQDFATVLNALPGLYEW
AN3371.1  	-DPTGR-----------TGLQRLPILPDIQTFVMRGAWNIMRDHQHWITLSQALPKVREW
          	 :  .               : :* :  ::*:: :* **::*. :.:  : .*** : **

MG09244.1 	HGTYNKPKSKSYLTMAEILPRLPPQLKQLHLYLEADYRRELSFPAYFVKVTNRLHFCQRL
NCU06056.1	HGSYSKPKSKSYLTMAEVLG-QPMSLSTLNLCIEGDYRRELSFPHYFNKVRTKVHFCSTL
FG09384.1 	QGSYSKPKSKSYITMAEFLPQLPRHITNLSVCLESDYRREGVMPPFYSKVIQKTHICARM
AN3371.1  	HCSYAKPKIEGYETIASIFRRLPSSLLHVNISLEGFYSKDNSQNRWLGDGVNPPHLCRLL
          	: :* *** :.* *:*..:   *  :  : : :*. * ::     :  .     *:*  :

MG09244.1 	GLAALGKPGSPSIEHLSYTGRVCRTFFDV----LAQS-ADPRSTRLKSIDLTVKNCCRQV
NCU06056.1	AQAAA----LPSMEHFSYTGRVCKQFFID----LAHRLKDIRNPRLKSIDITVKNCCRNV
FG09384.1 	AETLP------FLEHFAYTGRVCHQFFD-----IATRLTDSRDYRLKTLDLTVKNCCRYN
AN3371.1  	GEVAP------RLESLTFTGKVCACLFESTKNSMAIRPDKSQKSKLKSLDLVVKTCCRDR
          	. .         :* :::**:**  :*  :..::*    . :. :**::*:.**.***  

MG09244.1 	QIWNEM-----GSGIMDVKFIAAFEALVLAGIKALKRLRALEYLRIRYVDLD1SPIPALN
NCU06056.1	AHWNES-----GSGITDMFFIKAFEALVLAGIRSLEKLTSVKYLRIRYVDLD1SPVPPLN
FG09384.1 	TSFHEA-----GSGIQDMGFIDAFERLVLSAIRSLEKLKGVVYLRIRFVDLD1SVLPPLN
AN3371.1  	KLYPALPFLDDFSGITNLNFIRAFEELVVASINGLQIHQKLEYMRIRFIDLD~SACPPLN
          	  :   .  .. *** :: ** *** **::.*..*:    : *:***::*** *  *.**

MG09244.1 	PYFILRRDGWCEGVWSDKIVRALQASRPEARFRDLSDIPSFIGFGKDGRVVISAES-MGK
NCU06056.1	PYFQLR-NGWASGVWSEVIVSELNRVRPSVRFEELSESFGEVGYSKDGRMVINPDFPRAR
FG09384.1 	PFFIMR-NGACSGVWSERILAEMGRVRPDVHFTELSESFGNIVYNKDGRMVITPEPPKTK
AN3371.1  	PYFQLI-GTKATGLWNEPILEALHEARPEAQFVKLTDGISPQYGNNN--QIVGAIYPRTR
          	*:* :  .  . *:*.: *:  :   **..:* .*::  .    .::   :: .  .  :

MG09244.1 	VTSFKLDNYRYLSAGGGFVVAA
NCU06056.1	ALSLKLSNYRLLSGGISISHSG
FG09384.1 	ITSLKLSNYRSLATGITIQ---
AN3371.1  	PLSIHASTYRIIADVPKH----
          	  *:: ..** ::
```
